# Sequencing-Based Analysis of the Bacterial and Fungal Composition of Kefir Grains and Milks from Multiple Sources

**DOI:** 10.1371/journal.pone.0069371

**Published:** 2013-07-19

**Authors:** Alan J. Marsh, Orla O’Sullivan, Colin Hill, R. Paul Ross, Paul D. Cotter

**Affiliations:** 1 Teagasc Food Research Centre, Moorepark, Fermoy, Ireland; 2 Alimentary Pharmabiotic Centre, University College Cork, Cork, Ireland; 3 Microbiology Department, University College Cork, Cork, Ireland; University of Utah, United States of America

## Abstract

Kefir is a fermented milk-based beverage to which a number of health-promoting properties have been attributed. The microbes responsible for the fermentation of milk to produce kefir consist of a complex association of bacteria and yeasts, bound within a polysaccharide matrix, known as the kefir grain. The consistency of this microbial population, and that present in the resultant beverage, has been the subject of a number of previous, almost exclusively culture-based, studies which have indicated differences depending on geographical location and culture conditions. However, culture-based identification studies are limited by virtue of only detecting species with the ability to grow on the specific medium used and thus culture-independent, molecular-based techniques offer the potential for a more comprehensive analysis of such communities. Here we describe a detailed investigation of the microbial population, both bacterial and fungal, of kefir, using high-throughput sequencing to analyse 25 kefir milks and associated grains sourced from 8 geographically distinct regions. This is the first occasion that this technology has been employed to investigate the fungal component of these populations or to reveal the microbial composition of such an extensive number of kefir grains or milks. As a result several genera and species not previously identified in kefir were revealed. Our analysis shows that the bacterial populations in kefir are dominated by 2 phyla, the Firmicutes and the Proteobacteria. It was also established that the fungal populations of kefir were dominated by the genera *Kazachstania*, *Kluyveromyces* and *Naumovozyma*, but that a variable sub-dominant population also exists.

## Introduction

Kefir is a fermented milk-based beverage. It is a viscous, self-carbonated, acidic drink, which contains a low alcohol percentage and is believed to have originated in the Caucasian mountains some 2000 years ago. The milk is fermented by a solid, cauliflower-like, polysaccharide matrix known as a kefir grain, which is reused to start subsequent fermentations. The grain is primarily composed of bacterially-produced kefiran [1], which contains within it a complex consortium of bacteria and yeast that work in symbiosis to ferment the milk [2].

The microbial composition of kefir and kefir grains is believed to vary depending on geographic, climatic and cultural conditions as well as the diversity of local species of wild yeasts and bacteria. Culture-based analyses suggest that bacteria constitute the majority, up to 90%, of the population in the grain [3]. Such culture-based studies have also revealed that the bacterial composition of kefir predominantly consists of the lactic acid bacteria (LAB) *Lactobacillus*, *Lactococcus*, *Leuconostoc* and *Streptococcus* as well as acetic acid bacteria from the genus *Acetobacter* [4], [5], [6]. Bacteria contribute to the production of lactic acid, which preserves the milk, and produces various antimicrobial and flavour compounds (e.g. acetaldehyde) in addition to other metabolites (e.g. extracellular polysaccharides), free amino acids and vitamins [7]. Other studies have revealed that the yeast component of kefir consists of *Kluyveromyces*, *Saccharomyces*, *Candida* and *Torulaspora* [3], [8], [9], [10], [11]. Other yeast which have less frequently been associated with kefir include *Pichia*/*Issatachenkia* [9], [12], *Brettanomyces*/*Dekkera* [8], [13], *Zygosaccharomyces* [4] and *Yarrowia* [10], while recent molecular-based studies have detected the presence of *Kazachstania* [14], [15], [16]. Yeasts perform the vital role of alcohol and carbon dioxide production in the milk, and produce metabolites thought to be important with respect to mouthfeel and taste [17]. Ultimately, following a 24 hour fermentation, culture-based approaches indicate that lactococci/streptococci are present at 10^8^–10^9^ ml^−1^, *Leuconostoc* at 10^7^–10^8^ ml^−1^, acetic acid bacteria at 10^5^–10^6^ ml^−1^, lactobacilli at 10^5^–10^6^ ml^−1^ and yeasts at 10^6^–10^7^ ml^−1^ [18], [19].

Despite the undoubted value of the aforementioned studies, culture-based analyses are limited by virtue of only detecting species with the ability to grow on the specific medium used. Thus, culture-independent techniques have the potential to provide a more accurate and in-depth analysis. Although culture-independent techniques such as Sanger sequencing [12], [16], [20], [21] and DGGE [14], [15], [22] have been employed to explore the kefir population, the application of high-throughput DNA sequencing to investigate such microbial ecosystems has been a particularly significant development. This strategy has been employed to study the microbial composition of a number of fermented food environments such as cheese [23], [24], fermented fish [25], [26], fermented vegetables [27], rice bran [28] and pearl millet slurry [29]. Indeed, high-throughput DNA sequencing was also recently utilised to gain a more comprehensive understanding of the bacterial population of one Irish kefir grain and milk, and three Brazilian kefir grains [30], [31].

The benefits of gaining a better appreciation of the microbial composition of kefir and kefir grains relate to the fact that the history of kefir has long been linked to its purported health benefits. Preliminary studies have shown kefir to reduce lactose intolerance symptoms, stimulate the immune system, lower cholesterol, and to have antimutagenic and anticarcinogenic properties [7]. It is thus unsurprising that, as a functional dairy food, kefir has become the focus of increased study in recent years. While some of the health benefits thought to be derived from the consumption of kefir may be associated with the biochemical changes that occur within the milk, such as the production of organic acids, bioactive peptides etc., the microbial species present may also have health-promoting attributes. Notably, genera to which many strains with health-beneficial or probiotic properties are assigned, such as *Lactobacillus*, *Bifidobacterium*, *Enterococcus*, *Bacillus* and *Streptococcus*, have been isolated from kefir in the past [20], [32]. From a fungal perspective, strains of the yeast *Saccharomyces boulardii* have been established to possess health-promoting properties in clinical trials [33], [34], [35]. Strains of *Saccharomyces cerevisiae*, as well as *Kluyveromyces lactis*/*Candida kefyr*, commonly associated with kefir, also show potential in this regard [36], [37], [38]. Conversely, however, *Candida kefyr* has been shown to cause oesphagitis in a patient with squamous cell carcinoma [39].

Aside from identifying potentially health-promoting populations, the commercialisation of kefir production could benefit from gaining a detailed understanding of the associated microbial populations. There is also a need to assess the heterogeneity of these populations across a large number of grains and, in particular, to employ molecular approaches to better characterise the associated yeast populations. In light of these requirements, the aim of this study was to use high-throughput sequencing techniques to provide in-depth analysis of the microbial consortium of 25 distinct kefir grains and milks obtained from a variety of different sources in order to minimise any geographic bias that might influence the floras. This study represents the first occasion upon which this technology has been applied to such an extensive number of kefir samples and is the first study of its kind to reveal the fungal component of kefir.

## Materials and Methods

### Culture Maintenance

9 Irish kefir grains were recultured from −80°C storage within the Teagasc Culture Collection by fermenting in 10% reconstituted skimmed milk (RSM), which had been sterilized at 115°C for 15 mins. These were originally acquired from housewives across the country [18], and for the purposes of this study were designated IR1, 2, 3, 4, 5, 6, 8, 9 and 10. An additional 16 grains were obtained from individual and commercial suppliers from a number of different locations (Table S1), and cultivated under uniform conditions. Samples from the United Kingdom were designated UK1 to UK5 and samples from the United States were designated US1, 2, 3 and 5. Other kefir grains were sourced from Spain (Sp1), France (Fr1), Italy (It1), Canada (Ca1) and Germany (Ger1 and Ger2). Cultures were maintained at room temperature and inoculated into fresh milk 3 times per week, for a minimum of 4 months prior to extraction.

### Metagenomic DNA Extraction

100 mls of 10% RSM was inoculated with 1 g of kefir grain and fermented at 25°C for 24 hours, the time at which kefir is most frequently prepared. To extract DNA from the kefir, 1.8 mls of fermented milk was centrifuged to generate a pellet which was suspended in 450 ul of lysis buffer P1 from the Powerfood Microbial DNA Isolation kit (MoBio Laboratories Inc, USA). The resuspended pellet was subjected to enzymatic digestion with enzymes mutanolysin (100 U/ml) and lysozyme (50 µg/ml) at 37°C for 1 hour, followed by proteinase K (250 µg/ml) digestion at 55°C for 1 hour. Extraction was optimised with a 10 minute 70°C incubation [40] prior to mechanical lysis using the Qiagen TissueLyser II (Retsch**®**). The Powerfood Microbial DNA Isolation kit was then used as per the manufacturer’s instructions. Pure DNA was eluted in HPLC grade sterile water. DNA from kefir grain was isolated using a modified phenol-chloroform-based extraction procedure [22].

### DNA Amplification and Pyrosequencing

Metagenomic DNA extracts were used as a template for PCR amplification, with BioMix red (Bioline) which has a reported error rate of 2×10^5^ errors/bp [41]. PCR amplification of the V4–V5 variable region (408 bp) of the 16S rRNA gene was performed using the universal primers V1 (5′-AYTGGGYDTAAAGNG) and V5 reverse (5′-CCGTCAATTYYTTTRAGTTT) to facilitate an investigation of the bacterial component of the microbial populations [42]. Unique multiplex identifier adaptors, 8 bp in length, were attached between the 454 adaptor sequences and the forward primers to facilitate the pooling and subsequent differentiation of samples [43]. Tagged universal primers were also used to amplify fungal DNA from the variable ITS-1 rRNA region [44]. In this instance the forward primer ITS1F (5′-CTTGGTCATTTAGAGGAAGTAA) and ITS2 reverse (5′-GCTGCGTTCTTCATCGATGC) generated PCR products of *circa* 410 bp. The PCR conditions used for 16S amplification were 94°C denaturation for 2 min, 35 cycles of 94°C for 1 min (denaturation), 52°C for 1 min (annealing) and 72°C for 1 min (extension) followed by a final 72°C for 2 mins. The PCR conditions used for ITS amplification were 94°C denaturation for 4 min, 35 cycles of 94°C for 30 seconds (denaturation), 50°C for 1 min (annealing), and 72°C for 1 min and 30 seconds (extension). A final annealing step of 72°C for 10 mins was performed. All DNA was subject to a 10 min hotstart at 94°C prior to PCR amplification. Amplicons generated from three PCR reactions/template DNA were pooled and cleaned using the Agencourt AMPure**®** purification system (Beckman Coulter Genomics, Takeley, UK). Purified products were quantified using the Nanodrop 3300 Fluorospectrometer (Thermo Scientific) and the Quant-iT™ Picogreen**®** dsDNA Assay kit (Invitrogen). Equal concentrations of 16S or ITS amplicons were pooled, AMPure cleaned and assessed by an Agilent 2100 Bioanalyser (Agilent Technologies) to determine purity and to ensure the absence of primer dimers. Sequencing of the 16S rRNA V4–V5 and ITS1 rDNA amplicons was performed using a 454 Genome Sequencer FLX Titanium System (Roche Diagnostics Ltd) at Teagasc Food Research Centre, Moorepark, according to 454 protocols.

### Pyrosequencing Data Analysis

Raw sequences were quality trimmed and filtered using the Qiime Suite of tools [45]; any reads not meeting the quality criteria of a minimum quality score of 25 and sequence length shorter than 150 bps for 16S amplicon reads and 200 bps for ITS amplicon reads were discarded. The maximum homopolymer limit was increased to 10 for ITS amplicons as ITS sequences are known to harbour long homopolymer runs. Trimmed fasta sequences were assessed by BLAST analysis against the SILVA database (version 100) for 16S reads [46]. The ITS-1 specific database, ITSoneDB, was used to BLAST all ITS sequences [47]. BLAST outputs were parsed using MEGAN [48] with a bit-score of 86 was employed for 16S ribosomal sequence data, and a bit-score of 35 was used for ITS sequence data. The QIIME suite of programs was used to calculate alpha diversity including Chao1 richness, Shannon diversity, Simpson index, Phylogenetic Diversity and Observed species [45]. Sequencing depth was estimated using rarefaction analysis. QIIME was also used to generate weighted UniFrac, unweighted UniFrac and Bray-Curtis distances matrices. Principal Co-ordinate Analysis plots based on these distance matrices were generated with Qiime and visualised using King [49]. Statistically significant differences between the combined kefir grains and combined fermented milks were determined by the non-parametric Mann-Whitney test using the Minitab**®** statistical package. Reads were deposited in the SRA database under the accession number ERP002650.

## Results

### The Bacterial Population of Kefir Milk is More Consistent and Less Diverse than that of the Corresponding Grains

Post-quality filtering, 106,235 and 136,815 reads for 23 grain and the corresponding 23 milk samples, respectively remained, equating to an average of 4,619 reads for each grain sample and 5,949 reads per milk sample.

Chao1 values (reflective of OTU/species richness), Shannon and Simpson indices (to determine species diversity) as well as the Phylogenetic Diversity and Observed Species numbers were all calculated (Table S2). Rarefaction curves, calculated at 97% similarity, are approaching parallel to the x-axis for all samples, indicating sufficient reads were obtained to adequately assess the population (Figure S1). Box-plot analysis suggests that the bacterial population in kefir milk is generally less diverse than that present in the kefir grains (Figure S2), where the median value (black bar) for milk was lower in all metrics, with the exception of the Shannon index. The only significant difference between the grain and milks was in Phylogenetic Diversity (*p*<0.001).

**Figure 1:**
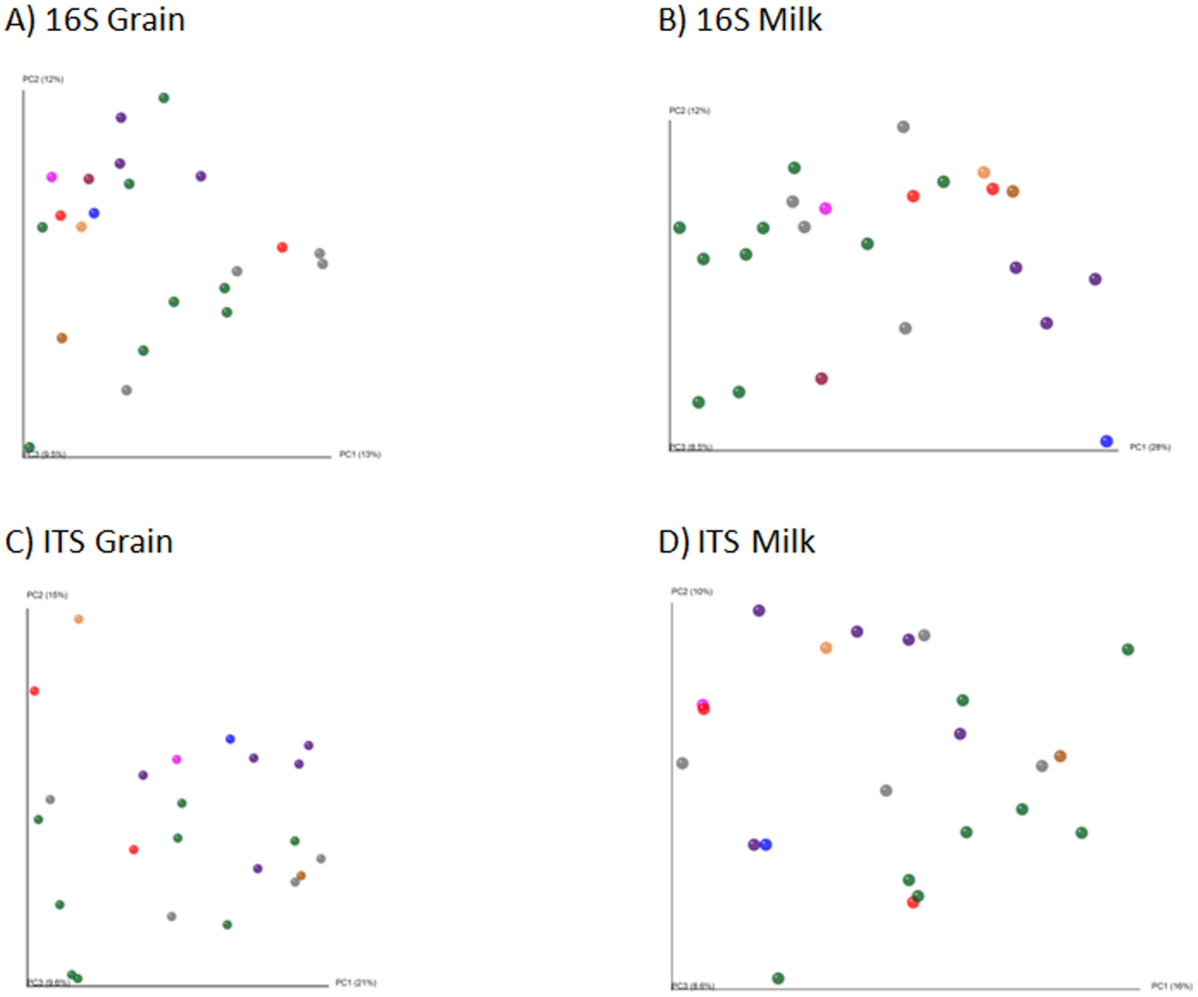
Principle Coordinate Analysis (PCoA) plots, based on unweighted UniFrac distance matrices, show the diversity within bacterial populations from kefir grains (A) and kefir fermented milk (B) and fungal grain (C) and milk (D) populations. Green = Irish kefir, Orange = Belgian kefir, Light Brown = Spanish kefir, Red = German kefir, Grey = US kefir, Pink = Italian kefir and Purple = UK kefir.

**Figure 2:**
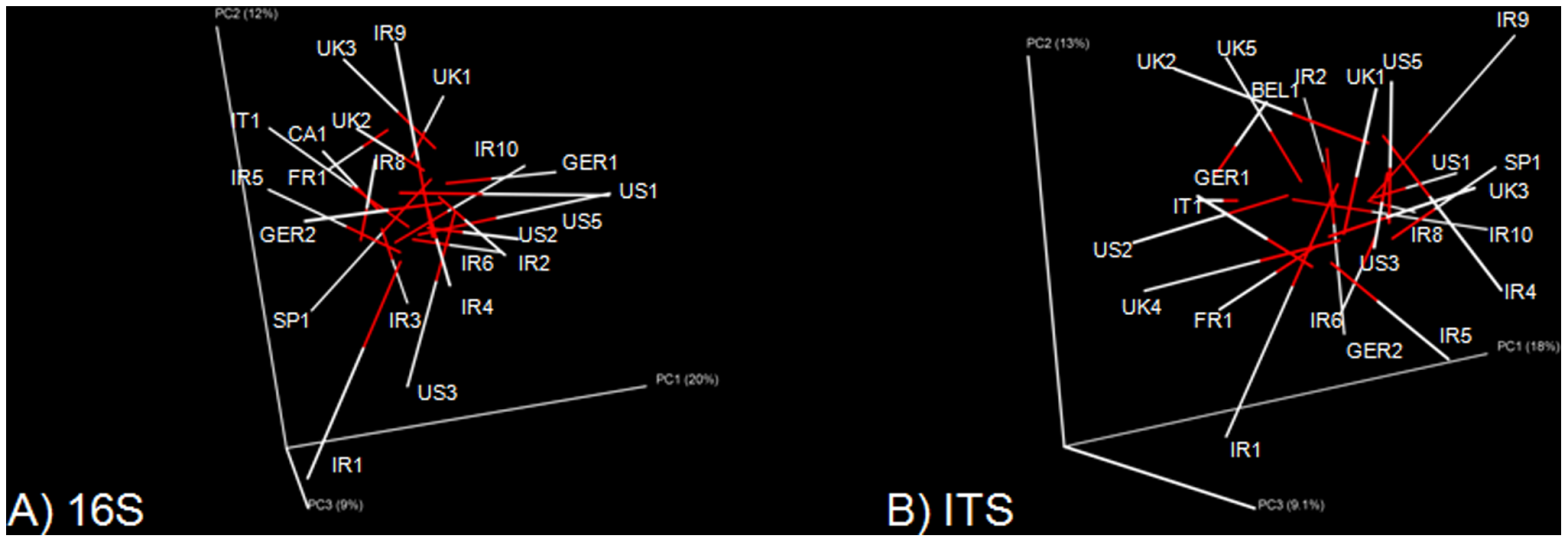
Procrustes imaging of unweighted UniFrac distance matrices highlight the diversity amongst the 16S bacterial component (A) and fungal component (B) of the different kefir samples. The two different sample types are linked with a bar (white represents grain flora; red represents milk flora). The direction of each axis is arbitrary.

Principal Co-ordinate Analysis plots were generated based on the unweighted UniFrac distance matrix (Figure 1AB), the only tree-based metric. From this analysis, it was evident that there was no clustering amongst kefir populations from different countries (Figure 1AB), and correlated with the other distance matrices (data not shown). Procrustes analysis indicated that the ordinations of kefir and kefir grains were not related to each other (*M* ^2^ = 0.924, *p = *0.644, Figure 2A). The similarities between kefir grain communities were not the same as the similarities between kefir communities.

### The Alpha Diversity of Fungal Populations in Kefir Milks and Grains Vary but the Beta Diversity of Kefir Grains is Greater than that of Milks

Post quality filtering a combined total of 118,879 and 118,976 reads corresponding to 23 grain and the corresponding 23 milk populations, respectively, were generated. This equated to an average read number of 5,167 and 5,173 per grain and milk sample, respectively.

Alpha diversity values established that there is a naturally low diversity in the kefir grains and milks (Table S3). Box-plot analysis of Chao1, Observed Species and Phylogenetic Diversity indices suggest diversity is greater in the kefir milk than in the grains (Figure S3). However, statistical difference between the two was limited to Phylogenetic Diversity (p<0.001). Rarefaction curves are approaching parallel to the x-axis for all samples, suggesting a sufficient depth of sequencing (Figure S4).

To measure beta diversity, Principal Co-Ordinate Analysis Plots were generated based on unweighted UniFrac distance matrices (Figure 1CD), but no clustering was evident. Procrustes analysis of the two PCoAs again shows that the similarities between the kefir grains and kefir milks were not the same, with respect to the fungal populations (*M* ^2^ = 0.855, *p* = 0.139, Figure 2B).

### The Kefir Grains and Associated Kefir-fermented Milks are Dominated by a Relatively Small Number of Bacterial Genera

Four bacterial phyla were detected in the kefir grain. These were the Actinobacteria, Bacteroidetes, Firmicutes and Proteobacteria. Of these, the Bacteroidetes were not identified among the milk bacteria, and were found in only 9 grains. Across both the grains and milks, the two dominant phyla were the Firmicutes and the Proteobacteria. Indeed most grain samples contained a majority (>50%) of Firmicutes, with the exception of Ir6, which possessed 69.14% Proteobacteria. Proteobacteria were not detected in grains Ca1, Ir9 or UK3. Among the milk samples, Ir1, Ir5, Ir10, US1 and Ir8 were also unusual by virtue of containing a bacterial population dominated by Proteobacteria, which in the case of Ir8, was as high as 90.4%. Milks corresponding to Fr1 and UK3 lacked Proteobacteria. No consistent shift (increase or decrease) in Proteobacteria populations from kefir grain to kefir milk was evident (Table S4; Table S5). Bacteria corresponding to the phylum Actinobacteria were detected in only two grains, Ir9 (5.87%) and UK2 (0.24%). The relatively high percentage of Actinobacteria in Ir9 may explain why the corresponding kefir milk was the only sample in which Actinobacteria were detected (0.26%). There was a significantly greater abundance of unassigned phyla among the total grains than the total milks (*p*<0.001).

At the family level, the greater bacterial diversity (in terms of number of different families) within the grain is evident. Only five families of bacteria were detected in the milk whereas twelve were identified in the grain samples (Tables S4–S5). The grains were predominantly composed of *Lactobacillaceae*, which accounted for >50% of the populations in all but grain Ir6. The other major family were the Proteobacteria-associated *Acetobacteraceae*. Other families detected were *Streptococcaceae* (19 grains), *Leuconostocaceae* (4 grains), *Lachnospiraceae* (16 grains), *Ruminococcaceae* (8 grains), *Bifidobacteriaceae* (2 grains), *Clostridiaceae* (2 grains), *Propionibacteriaceae* (2 grains), *Bacteroidaceae* (2 grains), *Enterococcaceae* (1 grain) and *Rikenellaceae* (1 grain) (Table S4). Among the other families, *Streptococcaceae* were detected in 19 of the 23 grains with the highest proportions found in UK2 (5.12%). *Leuconostocaceae* were found in only four of the grain samples (Bel1, 0.31%; Fr1, 0.13%; UK1, 0.29%; UK2, 0.51%). *Lachnospiraceae* were found in 16 grains from highest abundance in Ir9 at 0.51%, to lowest in US2 at 0.09%. *Ruminococcaceae* were found in 8 samples, from a high of 8.21% in Bel1 to a low of 0.08% in UK2. *Bifidobacteriaceae* were present in only 2 grains (0.81% in Ir9, and 0.10% in UK2), as were *Clostridiaceae* (Ger1, 0.39% and US2, 0.12%), *Propionibacteriaceae* (Ir9, 4.94% and UK2, 0.13%) and *Bacteroidaceae* (UK2 and UK3, 0.08%). *Enterococcaceae* (Ir9, 0.22%) and *Rikenellaceae* (US2, 0.07%) were present in only one grain each. The bacterial populations within the milks were dominated by *Streptococcaceae*, which were found at greater proportions in the kefir milks than in the grains (*p*<0.001), and form the dominant population (>50%) in 13 samples. Ir3, Ir8 and US1 were notable exceptions by virtue of containing 10.16%, 2.87% and 10.91% *Streptococcaceae*, respectively. In its place, Ir3 has the highest proportions of *Lactobacillaceae* at 60.51%, whereas Ir8 and US1 had the two highest proportions of *Acetobacteraceae* with 90.41% and 77.06%, respectively. However, in general, proportions of *Lactobacillaceae* were significantly lower in the milks than in the corresponding grains (*p*<0.001). The overall average proportion of *Acetobacteraceae* did not change significantly from the grains to the corresponding milks despite the fact that large increases were evident in same cases (i.e. the aforementioned Ir8 and US1 as well as Ir1 and Ir5). Proportions of *Leuconostocaceae* were detected in all kefir milk samples (in contrast to just 4 grain samples), reflecting a significant overall increase (*p*<0.001). *Propionibacterineae* was found in a single milk sample, Ir9, at 0.22%, which is a reduction from the 4.94% in corresponding grain. The proportions of unassigned reads were <1% in almost all grain and milks, with the exception of 1.02% in the grain of Ca1 (Table S4; Table S5).

**Figure 3:**
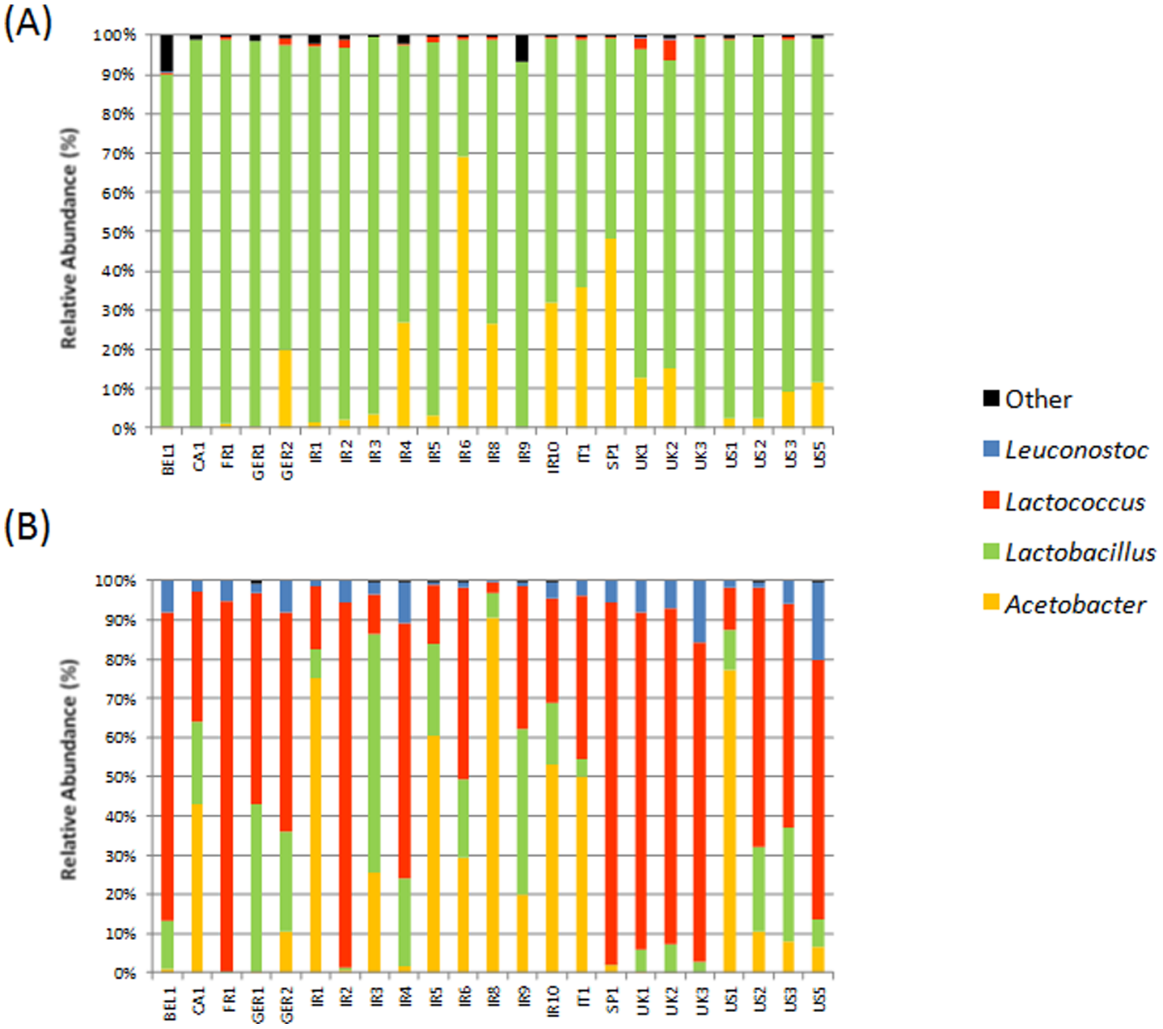
16S phylogenetic composition of the bacterial component of the kefir grain (A) and kefir fermented milk (B) at genus level.

The distribution pattern at the genus levels closely resembles that observed at family level, with one genus frequently corresponding to all reads assigned to that family (Figure 3). *Lactobacillus* (*p*<0.001) is the dominant genus in the grain with proportions of *Lactococcus* and *Leuconostoc* being significantly higher in the kefir milks (*p*<0.001). Once again, the differences in proportions and distribution of *Acetobacter* (of family *Acetobacteraceae*) in the grain and milk were numerically, but not statistically, different.

### ITS Sequencing Provides a Detailed Insight into the Fungal Composition of Kefir Grains and Associated Kefir-fermented Milks

The only fungal phylum assigned in the grain was Ascomycota, the largest phylum of the fungal kingdom. Ascomycota were also shown to dominate within the kefir milk, ranging from a high of 100% in Ger1 to a low of 89.38% in Ir10 (Table S6; Table S7). Basidiomycota, the other phylum belonging to the subkingdom Dikarya, was found in 9 milk samples at relatively low read numbers. 9 of the milk samples also harboured trace amounts of uncultured fungi. The lower diversity in the grain is again evident at the family level where all but one sample (Sp1) contain >99% *Saccharomycetaceae*. The overall average proportion of *Saccharomycetaceae* is significantly lower in the milks (*p*<0.001), but still correspond to >99% of reads in 16 of the 23 samples. The fungal composition of kefir milk Sp1 was unusual by virtue of containing 34.27% *Pichiaceae*. In contrast, the next highest proportion of *Pichiaceae* was 0.48% (in milk UK3). Other fungal families detected in both the kefir milks and grains were *Davidiellaceae* and *Trichocomaceae*. *Herpotrichiellaceae*, *Teratosphaeriaceae*, *Valsaceae*, *Debaryomycetaceae*, *Phaffomycetaceae*, *Malasseziaceae*, *Bondarzewiaceae*, *Dermataceae*, *Pezizaceae*, *Ganodermataceae*, *Tricholomataceae*, *Tremellomycetes*. In addition, *Wallemiomycetes* were only detected in the milks whereas *Dothioraceae* were only detected in the grains.

**Figure 4:**
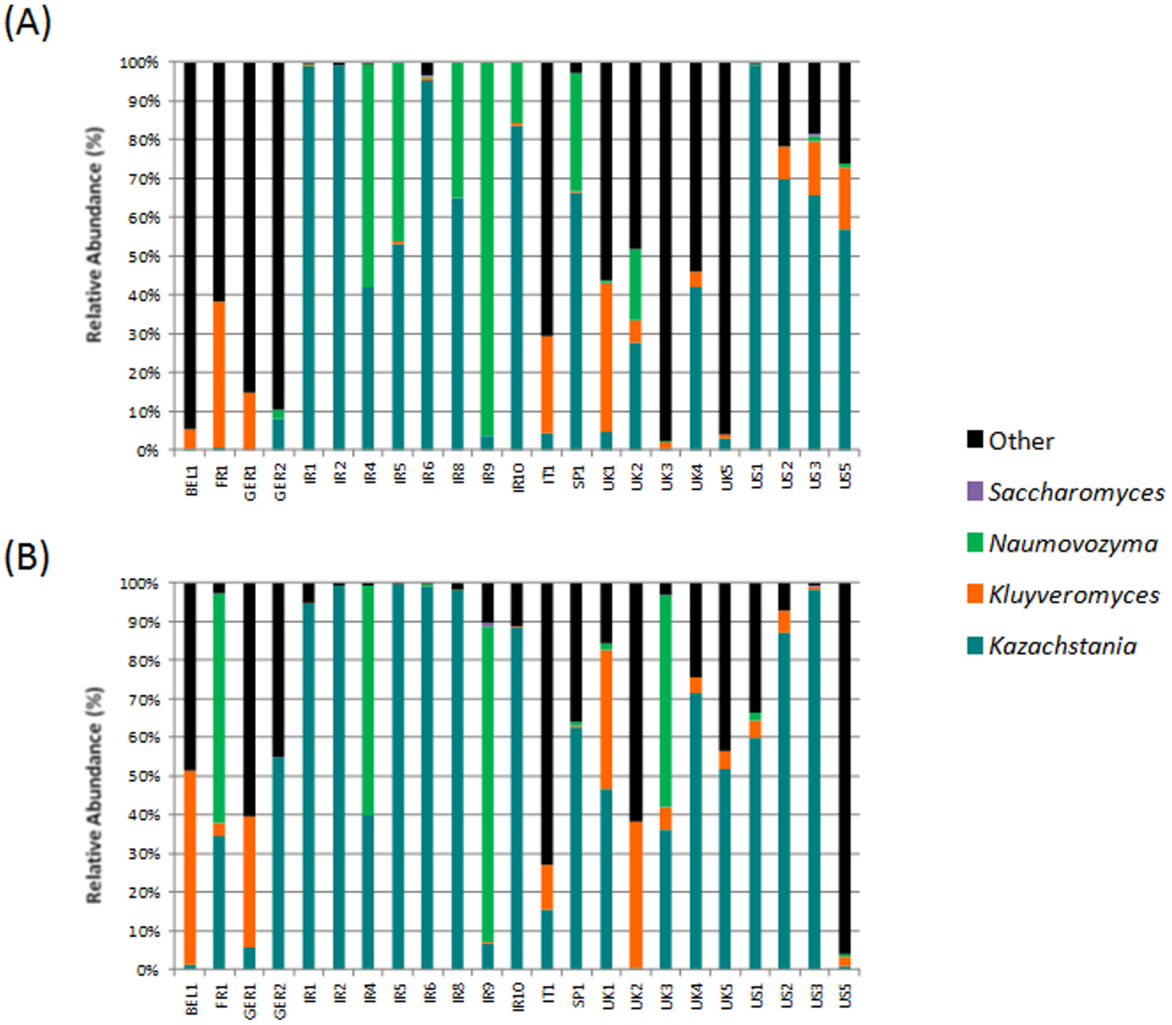
ITS phylogenetic composition of the fungal component of the kefir grain (A) and kefir fermented milk (B) at genus level.

The most common fungal genus across both the kefir milk and grains was *Kazachstania* (Figure 4). This genus was detected in all samples except kefir grain Ger1. Given that the corresponding milk contained *Kazachstania* at a proportion of 5.68%, it would seem likely that this grain did contain *Kazachstania* at levels below the limit of detection for this study. The proportions of *Kazachstania* were >50% in 11 of the grains and in 13 of the milks and was highest in grains Ir2 and US1 (99.40% and 99.25%, respectively) and the milks Ir2 and US3 (99.20% and 98.07%, respectively). In contrast, proportions were low in grains Bel1 and UK3 (0.24% and 0.39%, respectively) and milks UK2 and US5 (0.44% and 0.89%, respectively). *Naumovozyma* was the second most prevalent fungal genus, being present in 16 grains and 10 milk samples, accounting for 13.09% total grain reads, and 9.98% total milks reads. Proportions of *Naumovozyma* varied from being dominant in Ir9 (96.02%, grain; 81.87%, milk) and Ir4 (57.56%, grain; 59.41%, milk) to sub-dominant in Ger2 (2.46%, grain; 0%, milk) and US1 (0.18%, grain; 1.81%, milk), amongst others. Notably, although no *Naumovozyma* were detected in grain Fr1, this genus became dominant in the resultant kefir milk (59.3%), again suggesting the presence of *Naumovozyma* in the grain below the detection threshold. The third most commonly assigned genus was *Kluyveromyces*, which was detected in 17 of the grains and 18 of the milks, accounting for 7.6% and 7.32% of total grain and milk reads respectively. Although *Kluyveromyces* was present at a high of 50.16% in the milk of Bel1, this genus was more frequently present at sub-dominant proportions, with a detected low of 0.05% in the milk of Ir1. At genus level, many of the reads corresponding to the *Saccharomycetaceae* could not be reliably assigned. These corresponded to >50% of reads corresponding to grains Bel1, Fr1, Ger1, Ger2, It1, UK1, UK3, UK4 and UK5 and milks Ger1, It1, UK2 and US5. This is likely a result of such high similarity amongst ITS sequences that they cannot be reliably separated and assigned. Despite numerical differences in the proportions of the different fungal genera present in the kefir grains and milks, the only significant difference related to a higher proportion of *Dekkera* in the milks than in the grains (*p* = 0.004). The kefir milks also contained a larger number of different genera, often at trace levels, which were not detected in the corresponding grains. These included *Zygosaccharomyces*, *Wallemia*, *Eurotium*, *Microdochium*, *Cryptococcus*, *Teratosphaeria*, *Debaromyces*, *Cyberlindnera*, *Malassezia*, *Heterobasidion*, *Neofabraea*, *Peziza*, *Ganoderma*, *Mycena* and *Dioszegia*. *Penicillium* and *Aureobasidium* were each detected in only a single instance, i.e. in kefir grain Sp1 (0.13%) and grain UK3 (0.09%), respectively.

Unlike the 16S reads which are subject to a high level of sequence homology, the ITS reads were sufficiently dissimilar to enable assignment to species level. Table 1 shows the total number of different species identified and whether there has been a previous association with kefir. The population profile at species level strongly mirrors that at genus level. The most common species, *Kazachstania unispora*, was present in 20 grains and all milks. All reads from the *Kluyveromyces* and *Naumovozyma* genera were assigned to the species *Kluyveromyces marxianus* and *Naumovozyma castelli*, respectively (Table S6; Table S7). Although the *Saccharomyces* genus was identified in small amounts in a number of grains and milks, only those in Ir5 were assigned at the species level (to *Saccharomyces cerevisiae*).

**Table 1:** List of fungal species identified in the study, listed in teleomorph form with anamorph or synonym names and previous kefir association.

Species (Teleomorph)	Anamorph	Synonym	Previous Kefir Association
*Kazachstania barnettii*	N/A	*Saccharomyces barnettii*	No
*Kluyveromyces marxianus*	*Candida kefyr*	*Kluyveromyces fragilis, Candida pseudotropicalis*	Yes [14]
*Kazachstania unispora*	N/A	*Saccharomyces unisporus*	Yes [14], [31]
*Naumovozyma castelli*	N/A	*Saccharomyces castellii, Naumovia castellii*	No
*Saccharomyces cerevisiae*	*Candida robusta*	*Saccharomyces oviformis, Saccharomyces italicus*	Yes [11], [14]
*Davidiella tassiana*	*Cladosporium herbarum*	*Mycosphaerella tulasnei, Mycosphaerella tassiana*	No
*Penicillium s*p. *Vega* 347	N/A	N/A	No
*Pichia kudriavzevii*	*Candida acidothermophilum*	*Issatchenkia orientalis, Candida krusei,*	Yes [16]
*Pichia fermentans*	*Candida lambica*	*Candida fimetaria, Mycoderma lambica, Pichia sp.* AWRI 1271	Yes [9] [77]
*Dekkera anomala*	*Brettanomyces anomalus*	N/A	Yes [8]
*Dekkera bruxellensis*	*Brettanomyces bruxellensis*	*Brettanomyces custersii*	No
*Zygosaccharomyces lentus*	N/A	N/A	No
*Eurotium amstelodami*	*Aspergillus amstelodami*	*Aspergillus vitis*	No
*Wallemia sebi*	N/A	N/A	No
*Microdochium nivale*	N/A	*Fusarium nivale*	No
*Cryptococcus* sp. *Vega* 039	N/A	N/A	No
*Teratosphaeria knoxdaviesii*	N/A	N/A	No
*Cyberlindnera jadinii*	*Candida utilis*	*Pichia jadinii, Hansenula jadinii, Torula utilis, Torulopsis utilis*	No
*Malassezia pachydermatis*	N/A	N/A	No
*Heterobasidion annosum*	N/A	N/A	No
*Peziza campestris*	N/A	*Kimbropezia campestris*	No
*Ganoderma lucidum*	N/A	N/A	No
*Dioszegia hungarica*	N/A	*Bullera armeniaca, Cryptococcus hungaricus*	No

## Discussion

The study represents the most comprehensive investigation of the microbial population of kefir (both grains and milk) to date. This analysis was facilitated by high-throughput sequencing of 16S rRNA (bacteria) and, for the first time, ITS (fungi) amplicons, generated from a considerably larger collection of samples than has been employed heretofore. The number of reads compare well with previous studies i.e. Dobson *et al*. generated a combined total of 17,416 V4 16S rRNA (4,883 reads for the interior grain, 3,455 reads for the exterior grain and 9,078 reads for the milk fermentate; [30]) while Leite *et al*. generated a total of 14,314 16S rDNA reads (2,641, 2,690 and 8,983 reads for the three grains sequenced, respectively [31]). In each index, alpha diversity values were reflective of a naturally low diversity and a homogeneity between kefir samples, relative to other environmental analyses and rarefaction patterns were consistent with that of previous kefir studies [30], [31].

16S rRNA profiling revealed that the bacterial population of kefir milks tested is composed of Actinobacteria, Firmicutes and Proteobacteria, with Bacteroidetes also being detected in the grain. The kefir grains were dominated by *Lactobacillaceae*/*Lactobacillus*, establishing that this pattern, which was previously noted in high-throughput sequencing-based studies of a much smaller number of kefirs [3], [30], is consistent. In contrast, *Streptococcaceae* dominate in the kefir milk. More specifically, lactococci dominate as other genera from this family were not detected. This contrasts with a subset of previous studies in which *Streptococcus* species have been identified [3], [31], [50]. The next most common LAB were *Leuconostoc* sp.; *Leuconostoc* have been associated with kefir on a number of previous occasions [9], [19], [51], [52], but the data presented here reveals for the first time that the proportions of this genus increase considerably in the milk relative to the grain where they may significantly impact the sensory profile of kefirs. *Acetobacteraceae* (genus *Acetobacter*) were also identified as major components of the bacterial population of many grains despite having been identified in some [19], [53], but not all, previous kefir studies. However, given that kefir milks in which acetic acid bacteria were present at only very low levels (e.g. Bel1, Ir2, UK1) or were not detected (e.g. Fr1, UK3) underwent a successful fermentation, as determined by a reduction in pH and milk coagulation after 24–48 hours (data not shown), it may be that acetic acid bacteria are not strictly required for the fermentation process but rather contribute in some other way. Our further studies will focus on elucidating the precise contribution of specific populations on the consistency of kefir milk. The fact that *Lachnospiraceae* and *Ruminococcaceae* are present in several grains but not detected in the milk samples implies a poor ability to proliferate in the milk medium. *Bifidobacteria* were detected in two grains only (Ir9, 0.81% and UK2, 0.10%). These findings, coupled with previous studies, establish that bifidobacteria represent only a minor proportion of the kefir grain consortium. Furthermore, its poor endurance in the kefir milk suggests that it would need to be added in an encapsulated, or other such form, if kefir were to be employed as a vehicle for *Bifidobacterium* supplementation [54]. High-throughput sequencing also effectively unveiled the presence of a number of other rare populations in the kefir grains, which accounted for <1% of the overall population in most kefirs. Of these, *Faecalibacterium*, *Allistipes*, *Rickenellaceae*, *Allobaculum* and *Enterococcus* have not been identified in kefir previously and are typically associated with gut microbial populations. In contrast, *Pseudomonas* spp., identified in the grains of other high-throughput sequencing efforts in trace amounts, were not identified in these kefirs [30], [31].

After investigating the application of several ITS-specific databases, such as UNITE (http://unite.ut.ee/index.php), it was found that ITSoneDB, which consists of a comprehensive set of well-annotated and phylogenetically-classified ITS1 sequences derived form from Genbank and arranged on the NCBI taxonomy tree, gave the best assignment levels [47]. The composition of the kefir-associated yeast population has been the subject of some attention [2], [55] which has not been helped by nomenclature-related difficulties and a reliance, to date, on culture based investigations. The *Saccharomycetaceae* have a poorly defined group-specific morphology and such a basis for classification can lead to unreliable distinction of species from close relatives. Furthermore, many yeasts of the *Ascomycota* and *Basidiomycota* have sexual (teleomorphic) and asexual (anamorphic) states of reproduction, sometimes leading to classification of species under two names. It has been proposed that in 2013, fungi shall be known by only their teleomorph name, unless in extenuating circumstances [56], and thus this approach has been taken here. Examination of the literature highlights that *Candida kefyr* has previously been shown to constitute up to 90% of the yeast population in kefir milk [57] and has routinely been isolated from kefir [4], [57], [58]. Despite a significant presence in the ITS database, no *Candida* were detected in this study. Notably, however, a number of reads which displayed similarity with *C. kefyr* were instead assigned to the corresponding teleomorph, *Kluyveromyces marxianus*, by virtue of higher percent similarity. *Kluyveromyces marxianus* has previously been associated with kefir [10], [12], [16].

The dominant yeast detected in this study was *Kazachstania*, consisting of *Kazachstania barnetti* and *Kazachstania unispora. K*. *unispora* was previously known as *Saccharomyces unipsorus* [59], which has been identified in kefir [10], [12], [14], [15], [16] and has been associated with other fermented beverages [60], [61]. It would appear that *K. unispora* is particularly well adapted to the dairy environment as it is the most prevalent species, out-competing rival species including *K. barnetti*. This marks the first time *K. barnetti*, found in the grain but not in the milk, has been identified in a kefir environment. *Naumovozyma* is a genus that closely resembles *Saccharomyces* and *Kazachstania*, and the species identified here, *Naumovozyma castellii*, was reclassified from *Saccharomyces castellii* in the past [62]. Although it has not previously been linked with kefir, the only other species in the genus, *Naumovozyma dairenensis* (formerly *Saccharomyces dairenensis*) has been [63]. In contrast to the significant presence of the aforementioned fungal species, the relative absence of *Saccharomyces* is at first striking given its historical association with kefir. This is most likely reflective of the reclassification of *Naumovozyma* and *Kazachstania*. Despite this, it is notable that previous studies have suggested that *Saccharomyces cerevisiae* is quite common in kefir [3], [16], [52] whereas here the genus was detected in just three grains and three milks, and in trace amounts. It is possible that this genus is not as widespread as previous evidence suggested or may have been misassigned in previous studies. Alternatively, *Saccharomyces* may be more common in kefirs from geographic locations not included in this study. The origin of the grain may also have been significant with respect to the identification of *Pichia kudriavzevii* (previously known as *Issatchenkia orientalis*) at levels that were atypically high, relative to other samples, in the Spanish kefir (grain, 0.57%; milk, 34.27%). Notably, Latorre-Garcia *et al* identified *Issatchenkia orientalis* as one of the most representative species of Spanish kefir [12] and, until recently [16], it had not been found among non-Spanish kefir grains or milks. With respect to other species, it was also notable that *Torulaspora delbreuckii* was not detected in this study despite the fact that both it [10], [11] and its anamorph form, *Candida colliculosa*, have previously been detected in kefir [8]. There were also many instances whereby we identified species not previously detected in kefir milks, for instance while *Dekkera anomala* (anamorph: *Brettanomyces anomalus*) has been isolated from kefir [8], *Dekkera bruxellensis* (anamorph: *Brettanomyces bruxellensis*) has not been isolated from kefir before now (but has been found in traditional fermented Mongolean and Zimbabwean milks [64], [65]). Other species which had not previously been detected, but were present in lower abundance and few (often just one) milk sample(s) included *Cryptococcus* sp. *Vega* 039, *Zygosaccharomyces lentus*, *Penicillium* sp. *Vega 347*, *Wallemia sebi*, *Ganoderma lucidum*, *Cyberlindnera jadinii*, *Eurotium amstelodami*, *Heterobasidion annosum*, *Peziza campestris*, *Teratosphaeria knoxdaviesii*, *Dioszegia hungarica* and *Malassezia pachydermatis*. *Cryptococcus* and *Zygosaccharomyces* have been found in kefir before [4], but this marks the first identification of the respective species, *Cryptococcus* sp. *Vega* 039 and *Z. lentus*. *Cryptococcus* is a ubiquitous basidiomycotic yeast that was previously identified in a kefir that had been frozen and recultivated. This point is noted as the *Cryptococcus*-associated milks described in the current study resulted from two kefir grains, Ir8 and Ir9, which had been recultivated from −80°C storage. *Z. lentus* is considered a food spoilage organism associated with low-pH beverages and can grow at low temperatures [66]. *C. jadinii* is used in animal and human dietary supplements, and is a good source of vitamins, minerals, proteins and essential amino acids [67]. Despite not being isolated from kefir, it has been used to scale-up single-cell protein production using kefir [68]. Additionally, *E. amstelodami* is frequently isolated from bakers products [69]. *H. annosum*, *P. campestris*, *T. knoxdaviesii* and *D. hungarica* are all regarded as environmental fungi. *H. annosum* is the causative agent in the root and butt rot of pine trees [70], *Peziza* is associated with saprophytic cup fungal growth on rotten wood [71], *Teratosphaeria* have been described as eucalyptus pathogens [72] and *D. hungarica* has been shown to inhabit arbuscular mycorrhizal fungi [73]. *M. pachydermatis*, detected in Ir9, is a known pathogen which threatens neonatal infants and has been associated with domesticated canines [74]. Finally, in multiple samples (Bel1, Fr1, Ger1, Ger2, It1 and UK1-5), many *Saccharomycetaceae*-associated reads could not be assigned at the genus level and were designated as “other” (Figure 4). It is anticipated that as more fungal sequences are deposited, the species corresponding to these reads can be uncovered. The PCoA plots visualising the kefir microfloras do not show any obvious clustering amongst the different kefirs, showing the diversity between kefir populations regardless of their source.

The fact that natural kefir is capable of hosting several health-associated organisms suggests it could theoretically be altered to incorporate pre-established and certified probiotic strains, with minimal sensory impact. Indeed, the ultimate application of kefir may be as a potential delivery system for viable health-promoting organisms to the gut [75]. However, the fact that grains have yet to be produced from pure culture [76] suggests that there remains a lot to be understood regarding the population dynamics of kefir grains.

In conclusion, the study represents the most comprehensive investigation of the microbial composition of kefir grains and milks to date. It provides important information that may facilitate the reconstitution of kefir grains to create tailored kefir grains and milks while further investigation of the specific components identified can reveal their contribution to the kefir grain structure and the health-promoting aspect of the associated beverages.

## Supporting Information

Figure S1 **Rarefactions for the 16S kefir milk and grain Chao1 and Shannon indices where A = Grains and B = Milks.**
(DOC)

Figure S2 **Box plots of the 16S alpha diversity.**
(DOC)

Figure S3 **Box plots of the ITS alpha diversity.**
(DOC)

Figure S4 **Rarefactions of the ITS kefir milk and grain Chao1 and Shannon indices where A = Grains and B = Milks.**
(DOC)

Table S1 **Sources of kefir samples.**
(DOC)

Table S2 **16S alpha diversities.**
(DOC)

Table S3 **ITS alpha diversities.**
(DOC)

Table S4 **Relative abundances for the 16S grain.**
(DOC)

Table S5 **Relative abundances for the 16S milk.**
(DOC)

Table S6 **Relative abundances for the ITS grain.**
(DOC)

Table S7 **Relative abundances for the ITS milk.**
(DOC)
