# Supplementary material for: Sequencing-Based Analysis of the Bacterial and Fungal Composition of Kefir Grains and Milks from Multiple Sources
Source: PLoS One. 2013 Jul 19;8(7):e69371. doi: 10.1371/journal.pone.0069371 (PMC3716650; doi:10.1371/journal.pone.0069371)
Supplement: Figure S3 — Box plots of the ITS alpha diversity. (DOC) [file pone.0069371.s003.doc]

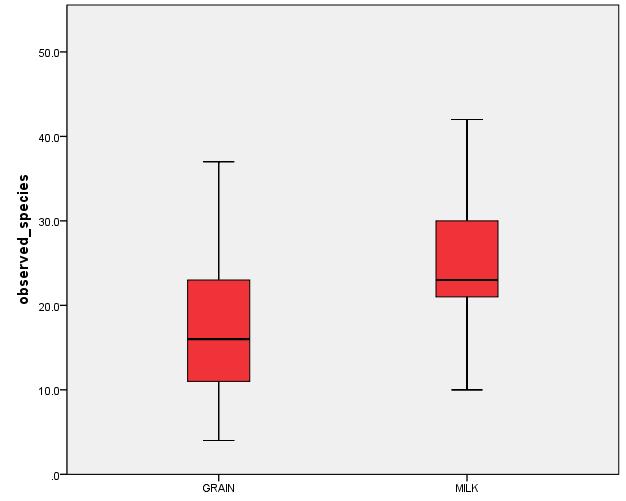

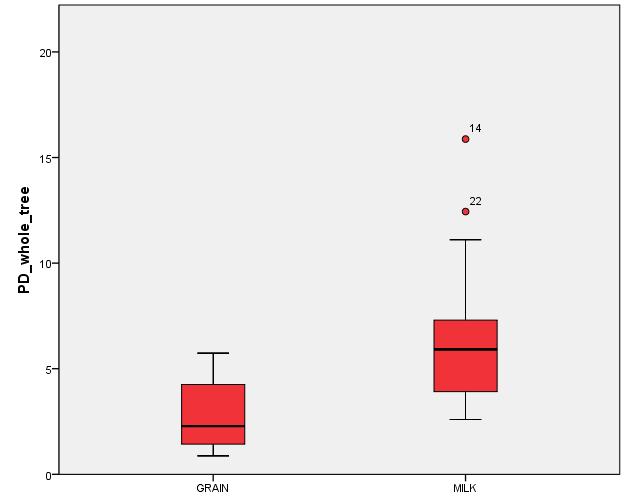

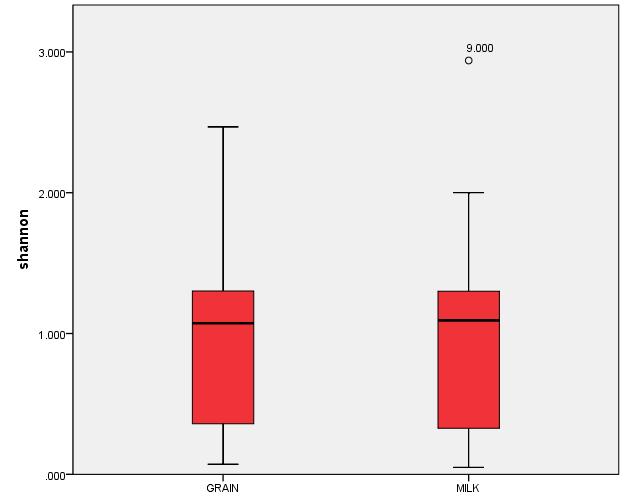

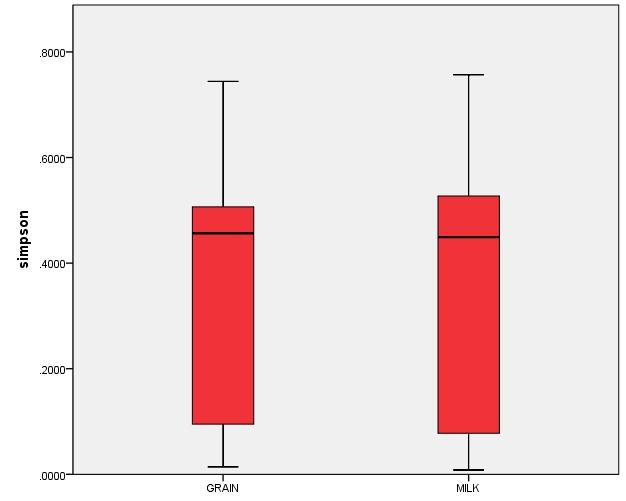

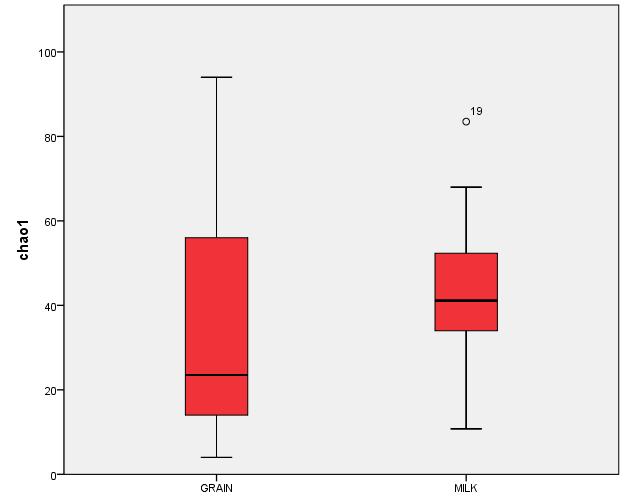


**Chao1**

**Shannon**

**Simpson**

**Observed Species**

**Phylogenetic Diversity**

**Supplemental Fig. 3**

Box plots of the ITS alpha diversity
