# Supplementary material for: Sequencing-Based Analysis of the Bacterial and Fungal Composition of Kefir Grains and Milks from Multiple Sources
Source: PLoS One. 2013 Jul 19;8(7):e69371. doi: 10.1371/journal.pone.0069371 (PMC3716650; doi:10.1371/journal.pone.0069371)
Supplement: Figure S4 — Rarefactions of the ITS kefir milk and grain Chao1 and Shannon indices where A = Grains and B = Milks. (DOC) [file pone.0069371.s004.doc]

**A) Grain**

**B) Milk**

**Chao1**

**Chao1**

**Shannon**

**Shannon**


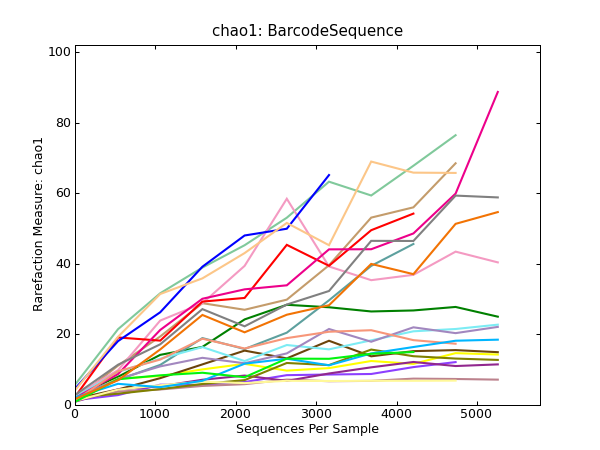

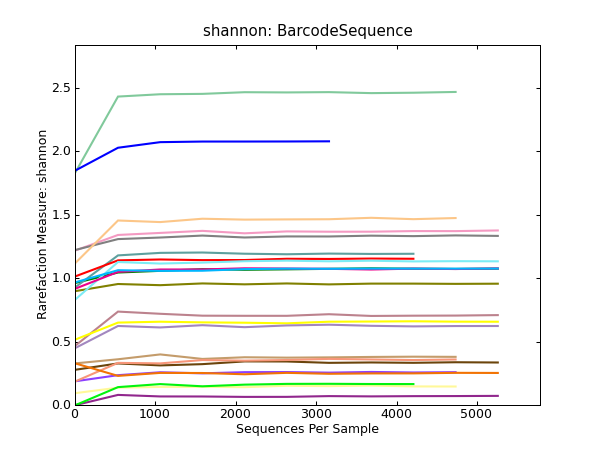

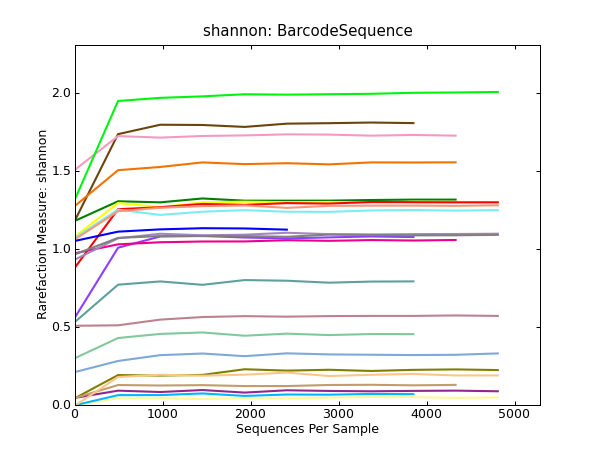

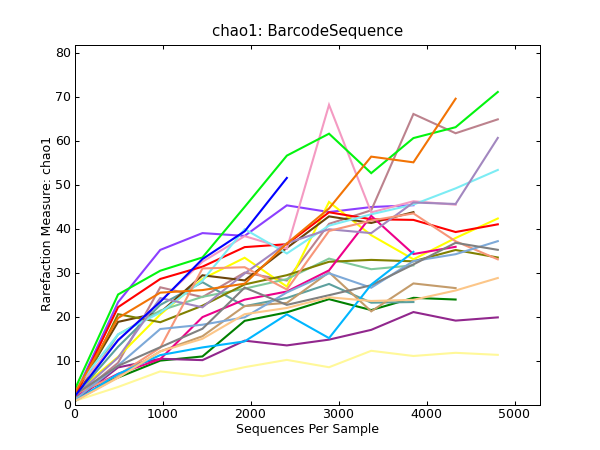


**Supplemental Fig. 4**

Rarefactions for the ITS kefir milk and grain Chao1 and Shannon indeces

A) = Grains B) = Milks
