# Supplementary material for: Sequencing-Based Analysis of the Bacterial and Fungal Composition of Kefir Grains and Milks from Multiple Sources
Source: PLoS One. 2013 Jul 19;8(7):e69371. doi: 10.1371/journal.pone.0069371 (PMC3716650; doi:10.1371/journal.pone.0069371)
Supplement: Table S2 — 16S alpha diversities. (DOC) [file pone.0069371.s006.doc]

**Supplemental Table S2**

16S Alpha Diversities
