# Supplementary material for: Sequencing-Based Analysis of the Bacterial and Fungal Composition of Kefir Grains and Milks from Multiple Sources
Source: PLoS One. 2013 Jul 19;8(7):e69371. doi: 10.1371/journal.pone.0069371 (PMC3716650; doi:10.1371/journal.pone.0069371)
Supplement: Table S5 — Relative abundances for the 16S milk. (DOC) [file pone.0069371.s009.doc]

**Supplemental Table S5 (Part 1)**

Relative Abundances for the 16S Milk

**Supplemental Table S5 (Part 2)**

Relative Abundances for the 16S Milk
